# Relative Preservation of Advanced Activities in Daily Living among Patients with Mild-to-Moderate Dementia in the Community and Overview of Support Provided by Family Caregivers

**DOI:** 10.1155/2012/418289

**Published:** 2012-06-28

**Authors:** Hajime Takechi, Atsuko Kokuryu, Tomoko Kubota, Hiroko Yamada

**Affiliations:** ^1^Department of Geriatric Medicine, Graduate School of Medicine, Kyoto University, Sakyo-ku, Kyoto 606-8507, Japan; ^2^Department of Social Welfare, Faculty of Social Studies, Doshisha University, Kyoto 602-8580, Japan

## Abstract

Little is known about the extent to which advanced activities of daily living among patients with dementia are preserved and how family caregivers of these patients support them in the community. In this cross-sectional assessment of pairs of patients with dementia and their family caregivers, we evaluated basic, instrumental, and advanced activities of daily living by comparing past and present status observed by caregivers with subjective estimations by patients with dementia. We also asked about ways in which support was provided by family caregivers. Thirty-nine pairs of patients with dementia and caregivers who presented to our memory clinic were interviewed. The mean age of patients with dementia was 75.3 ± 7.0 years, and Mini-Mental State Examination scores were 22.3 ± 3.4. We found relative preservation of advanced activities of daily living compared with instrumental activities of daily living. Caregivers provided instrumental, informational, and reminding support to patients with dementia. These findings may reinforce the concept of person-centered support of patients with dementia in the community.

## 1. Introduction

As the number of patients with Alzheimer's disease (AD) increase, social care costs that patients, their families, and society pay are expected to increase [1]. Although Banerjee and Wittenberg report various advantages of early diagnosis of dementia [2], including social cost reductions, the effects of pharmacological therapy are limited, as drug therapy induces only slight improvements in patients and caregivers' daily lives. In addition, caregivers often have limited social support [3].

Patients with AD are often unable to complete activities of daily living (ADL). This inability spans from basic ADL (BADL), such as clothing and bathing to instrumental ADL (IADL), such as shopping and food preparation, and advanced ADL (AADL), such as hobbies and working. The preservation of advanced activities is important to help people maintain their self-identity. Support and intervention for patients such as person-centered care and dementia care mapping, which includes understanding the patient's life history, individuality, and perspectives, are being widely accepted as an approach to deliver high-quality dementia care [4]. With this style of support, care workers recognize that the personality of the patients with dementia is concealed rather than lost, personalize the person's care and environment, offer shared decision-making, interpret behaviors from the viewpoint of the person, and prioritize the relationship with the patients as much as the care tasks [5]. This approach may reduce agitation and result in use of significantly fewer neuroleptic medications in nursing home residents [6, 7]. In addition, before a diagnosis is given and until family caregivers find formal support after a diagnosis is made, family members can provide the so-called person-centered care by themselves during daily caregiving because they know the patients well.

Compared with the late stage of dementia, patients and family tend to experience more difficulties in early- to middle-stage dementia with behavioral psychological symptoms of dementia (BPSD). Lövheim et al. revealed a higher prevalence of BPSD such as aggressiveness, wandering, restless behavior, hallucinatory symptoms, and depressive symptoms in middle-stage dementia and showed that persistent symptoms of passiveness, including apathy, often are more prominent in the later stage [8]. Maslow suggested a theory of human motivation from basic physical needs to self-actualization [9], and Buron recommended analyzing support needs of patients with AD along with Maslow's model to provide person-centered care [10]. When basic physical needs are satisfied, patients tend to have psychosocial needs, especially when the care is focused on the body or care tasks [11, 12]. Although support for patients in BADL and IADL to maintain minimal requirement of their daily lives has been discussed and is being developed, studies on support in early- to middle-stage dementia, and in particular in AADL, are scarce.

To provide better person-centered care from the early stage of dementia, person-centered support in AADL should be developed. In addition, this care should be given by formal caregivers, volunteers, and/or mutual aids in addition to family caregivers to reduce the burden of family caregivers in an ageing society and the rapid increase of patients. The aim of this study was to analyze declines in different types of ADL (BADL, IADL, and AADL) by comparing current and previous (around but before family noticed first symptoms of dementia) support needs in ADL retrospectively in community dwelling patients and to examine what kind of support family caregivers provided, especially in the early stages of dementia.

## 2. Methods

### 2.1. Participants

Community dwelling peoples with mild-to-moderate dementia and their family caregivers who regularly visited the memory clinic in Kyoto University Hospital for more than 6 months were invited to participate. To be included, the patient's Mini-Mental State Examination (MMSE) score had to be greater than 15, and they had to have a family caregiver, regardless of whether they were living together or not.

All participants were informed about the aim, risks, and benefits of the study, and consented to be included. This study was approved by the ethical committee of Kyoto University Hospital.

A total of 39 patients with AD (18 males and 21 females; mean age, 75.3 ± 7.0 years; mean MMSE score, 22.3 ± 3.4) and their family caregivers (12 males and 27 females) were included. The mean duration from the first visit at our hospital to the interview was 2.1 ± 1.4 months. The mean age of caregivers was 63.1 ± 13.8 years. The relationships to the patient were spouse, 23 (59.0%); child, 11 (28.2%); spouse of patient's child, 3 (7.7%); and other, 2 (5.1%). The diagnosis of AD was made according to the Diagnostic and Statistical Manual of Mental Disorders, 4th edition National Institute of Neurological and Communicative Disorders and Stroke, and the Alzheimer's Disease and Related Disorders Association [13, 14].

### 2.2. Survey Questions and Statistical Analysis

Interviews took place from September 2004 to October 2005 by clinical psychologists and a speech therapist who are skilled at dementia care. During an interview, all participants (39 pairs of patients and their family caregiver) were asked how much support the patient needed in ADLs at the time of the interview and in the past (around but before the onset of symptoms of dementia), and what kind of assistance family caregivers provided in daily lives. Patient's support needs in AADL and IADL were evaluated by the patient and caregiver separately. Also, patient's levels of BADL (toilet, feeding, dressing, grooming, physical ambulation, and bathing) were rated by caregivers using a 5-point scale from 0 (fully dependent) to 4 (independent) based on the Lawton scale [15]. Lawton's IADL (responsibility for own medications, ability to handle finances, mode of transportation, food preparation, shopping, ability to use telephone, housekeeping, and laundry) were also rated by patients and caregivers on a 4-point scale (0, does not do spontaneously; 1, needs extreme assistance and support; 2, needs a little assistance and reminding or does it imperfectly; 3, fully independent). Although Lawton and Brody developed a 2-point scale for these variables, we evaluated patient's IADL using a 4-point scale in this study to evaluate support needs more precisely.

We chose AADL items referring to Koyano's paper validating Tokyo Metropolitan Institute of Gerontology (TMIG) index where the highest two sublevels of competence among Lawton's seven sublevels are regarded as AADL [16]. We also chose items from leisure activities described by Baltes et al. where the activities of elderly was divided into obligatory activities (BALD/IADL) and leisure activities [17]. The 8 common AADL items interviewed in this study were selected two from TMIG index (reading a newspaper, giving advice to family), four from the study by Baltes (watching TV, taking a walk, care of a grandchild, socializing with others) and two activities (shopping on special occasions, participating in a meeting) were subjectively selected taking urban environment of the survey area and typical ability of old people there into consideration. We asked carefully to avoid patient's passive behavior in AADL, asking caregivers, for example, “Does he/she actively watch TV programs which he/she is interested in?” We divided AADLs into two categories: common AADLs that include intellectual and social activities common to many people, and leisure activities, which can vary in individuals depending on the person's character, interests, and routines. Leisure activities included those suggested by Baltes et al., such as gardening and reading [17]. In addition, patients and caregivers were asked to list as many activities and interests of patients as possible. For example, based on Baltes et al., interviewers asked “what has the patient been interested or stuck on these days?” Support needs for common AADLs were rated using the same 4-point scale used for IADL. Activities that the patient has not done since he/she was healthy were not taken into account in the analysis. In addition, caregivers provided concrete examples in which they provided daily assistance for patients in AADLs. Their support was classified using support categories suggested by House as follows [18]. Taking the patient to the place he/she needs to go, doing activities with the patient, and preparing for the activity were categorized as “instrumental support.” Giving specific advice to the patient was categorized as “informational support.” Praising or encouraging the patient was classified as “appraisal support,” and listening to the patient and staying beside the patient were classified as “emotional support.” In addition, “reminding support” such as reminding the patient of events and activities was added to this study.

Patient's declines in different types of ADL (BADL, IADL, and AADL) in addition to differences between caregiver's ADL evaluations and patient's self-evaluation were analyzed using the paired *t*-test. Caregivers were allowed to describe more than one type of support for a single activity, and the mean number of support categories for each activity was calculated. All data were analyzed using SPSS version 17.0. *P* values less than 0.05 were considered as significant.

## 3. Results

Table 1 shows background characteristics of participants. Compared to previous levels (before the symptom onset), BADL declined by 11.3% (±8.7), IADL declined by 57.4% (±19.5), and AADL declined by 46.4% (±20.0) based on caregiver's assessment. There was remarkable deterioration in IADL and AADL compared with BADL (*P* < 0.01, BADL versus IADL; *P* < 0.01, BADL versus AADL). The difference in decline between IADL and AADL was not significant. Among BADL, significant declines (*P* < 0.05) were observed in toileting, bathing, dressing, and grooming; no significant changes were seen in physical ambulation and feeding (Table 2).

There were significant declines in all IADL (*P* < 0.01) based on caregiver's assessment. Remarkable declines were observed in responsibility for own medications and handling finances. There was a wide range of decreases in previous IADL scores depending on the activities, because original levels largely varied depending on individuals; however, all changes were significant. In addition, significant declines were shown in all AADL.

 Patient's self-evaluation of current ADL levels was compared with caregivers' evaluation (Table 3). Although patients recognized their functional declines, they generally estimated their activity levels as much higher than objective observations provided by family caregivers. In IADLs, significant differences between patient's self-evaluation and caregiver's objective evaluations were shown in all eight activities. On the other hand, there were no significant differences between patient's self-evaluation and caregiver evaluations for the AADLs of taking a walk, watching TV, shopping on special occasions, and taking care of grandchildren. The level of inconsistency between the patient and caregiver was calculated for each IADL and AADL by the dividing patient's self-evaluation score by the caregiver score. Levels of inconsistency were larger for IADLs (1.98 ± 0.42) than for AADLs (1.38 ± 0.34, *P* < 0.05).

When focusing on the average number of activities that patients had continued and those that patients had quit since symptom onset, it was revealed that most patients could continue watching TV (89.8%) and shopping on special occasions (89.1%) as common AADLs. However, a remarkable number of patients quit reading newspapers (28.9%), participating in meetings (50%), and giving advice to the family (43.2%). Patients tended to cite traveling, gardening, hobby classes, and going to theaters as their leisure activities, although there were wide variations in activities. Patients seemed to be able to continue two leisure activities on average.

Table 4 shows how many types of support caregivers provided for each common AADL at the time of the interview. Most support was categorized as instrumental support (34.6% of total supports) and reminding support (38.0%). Caregivers sometimes provided emotional support (16.7%) and informational support (10.2%). In general, there was less appraisal support compared with other types of support. Instrumental, informational, and reminding support tended to be provided when patients went out such as for shopping and meetings. In addition, among leisure activities, caregivers were more likely to provide support for going out such as travelling and going to theaters. On the other hand, patients seemed to enjoy some activities (playing music, singing, taking care of pets, and gardening) without support.

## 4. Discussion

Although patients' BADL declined gradually, greater deteriorations were observed in AADL and IADL. We expected larger declines in AADL compared with IADL based on Maslow's hierarchy of motivations [9]. However, there was no significant difference between declines in AADL and those in IADL. There were significant gaps between patient's self-evaluation of ADLs and caregiver's evaluations. Reminding support, which seems to play an important role in encouraging patients in activities, instrumental support, and informational support, made up a large percentage of total support for AADL and leisure activities. Most support in AADL and leisure activities was provided for patient's going out.

From the viewpoint of complexity of activities and Maslow's hierarchical model, we hypothesized that AADL would be more damaged rather than IADL in dementia patients. In fact, Vriendt et al. describe that detecting subtle changes in AADL in addition to IADL can be more useful to detect early symptoms of dementia [19]. However, our study showed no significant difference between IADL decline and AADL decline. The reason why AADL decline was similar to that of IADL could be firstly because of the difficulty of capturing the levels of AADL in the manner to compare it accurately with IADL. Secondly, caregivers might tend to underestimate decline of patients' AADL, since it is assumed that patient's AADL could be maintained with little help such as reminding compared to IADL support. Thirdly, their leisure activities could be necessarily maintained because enjoying leisure activities is deeply connected to their character and self-identity, and no one can enjoy instead of the patient. Fourthly, we could not easily hypothesize that AADL would be damaged earlier compared to IADL in persons with dementia, because ability to perform AADL can be affected by not only cognitive function but also social and emotional factors which are deeply affected by patient's self-identities.

It seems to be easier to arrange support for BADL and IADL rather than AADL in patients, because there are less individual variations in how to provide this support. However, the importance of support in AADL and leisure activities cannot be overemphasized from a viewpoint of person-centered care [4, 5]. Cohen-Mansfield et al. suggested that hobbies/leisure activities can be one of four important domains of self-identity (hobby/leisure activities, professional role, family role, and personal attributes) [20] and showed that encouraging activities that stimulate patients' identity roles could increase their interest, pleasure, and activity involvement, as well as reduce their agitation in residential care settings [21].

Unfortunately, support in AADL and leisure activities is often overlooked or omitted from public social services, because lack of support in AADL or leisure activities is not life threatening, whereas patients cannot survive without support for eating or toileting. In fact, support covered by Japanese Long-Term Care Insurance (LTCI) is limited to essential housekeeping and physical care. The system does not allow home helpers to support patient's leisure activities or AADL [22].

This study revealed that family caregivers often provide instrumental support, such as taking a patient to shopping or to activities, in addition to reminding support, including informing patient of events and activities. These kinds of support could help patients maintain independence and autonomy and should be included as part of dementia care. Providing these types of support is not time consuming if the caregiver knows the patient's lifestyle and interests. However, this is not usually the case for outside caregivers, and thus providing this type of support is not easy for several reasons. First, caregivers need to learn additional skills to help them gain information about the patient's life history and individuality. Second, learning about individual patients can be time consuming and it is difficult for service providers to charge for this seemingly “non-medical” service such as reminding, which can be finished within minutes.

Activities for patients with dementia in formal care settings, including day care and short-term care, should be person-centered as well. Activities should be beneficial to patients and suited to individual interests and preferences. However, activities for patients with dementia in formal care settings and the efficacy of these activities have not been well studied, because these short-term care services were originally started primarily to provide relief for family caregivers. In fact, uniform exercise and recreation activities are often offered during formal care, and the effects of institutional respite on care recipients, including ADLs, BPSD, and cognitive function are still inconclusive [23–25].

To improve person-centered dementia care in the community, it is important for care providers to reconsider care skills and service provision by knowing what kind of support family caregivers provide in daily care. Furthermore, person-centered care can be strengthened by supporting family caregivers, who know the person's life history and preferences. In a longitudinal study, Burgener and Twigg showed that care recipient's quality of life (QOL) can be affected by caregiver factors such as caregiver distress [26]. Family caregivers of patients should be supported socially, psychologically, and economically by society and the community, although differences in attitudes to family caregiving may vary by culture.

Gaps between patient's self-evaluation and caregiver evaluation in ADLs (especially IADL and AADL) indicate the need to ask caregivers as well as patients about declines in ADL, because patients might not be aware of small declines or pathological changes. Service and care providers should listen to caregiver's evaluations along with patient's self-evaluation when arranging support, so that the patient's activity levels are evaluated appropriately, and they can be supported in activities that stimulate their sense of identity. As Cohen-Mansfield et al. suggested, the goal of support should be individual patient's self-actualization and maintaining identity, not only supporting them in essential BADL [21]. Regarding relative preservation of evaluated status of AADL by patients compared to that of IADL, we guess that the patients could be psychologically reluctant or shameful to admit their loss of IADL functions by themselves compared to AADL, because IADL may require more functional element obligatory to daily life than AADL, resulting in denial of the present real IADL status of them. Another reason may come from caregivers' support for AADL below the surface which could maintain the patients' AADL without their awareness.

Limitations of this study include sampling bias and recall bias: participants were recruited in only one clinic in one university hospital in Japan, and data (previous levels of ADL) were collected retrospectively at one interview. Longitudinal observation studies are needed to reveal if family support in AADL can be helpful in maintaining patient's QOL. In addition, similar studies should be undertaken worldwide to reveal regional differences and cultural differences in family caregiving and support for patients with dementia. Another limitation is that we could not show the evidence of the benefit of caregiver's support on patient's QOL in this study. We asked about it in our interview using a visual analogue scale. The analysis did not show any significant associations between them even after controlling for care recipients' age and dependencies in BADL/IADL, possibly because there are a large number of factors which can affect patient's QOL: patient's dependency level and severity of dementia, amount of caregiver support, patient's lack of awareness of being helped, relationships between patient and caregiver, and so on (data not shown).

## 5. Conclusion

This study revealed that family caregivers of patients with AD provide various supports in patient's AADL and leisure activities, which could play an important role in maintaining the identities of patients. It is difficult to assess levels of AADL in patients with dementia because there are individual variations in preferred activities and daily routines. However, knowing the type of support provided by family caregivers may be helpful when support for the patients with dementia is going to be provided by mutual aids in communities, volunteers, or additional care workers in the future.

## Figures and Tables

**Table 1:** Demographics of patients and caregivers.

	Patients (*n* = 39)	Caregivers (*n* = 39)
Age (y)	75.3 ± 3.4	63.1 ± 13.8
Female (%)	53.8	69.2
MMSE score	22.3 ± 3.4	
Family relationship		
Spouse		23
Children		11
Daughter-in-law		3
Other		2

MMSE: Mini-Mental State Examination.

**Table 2:** Differences in ADL activities of patients; past and present, based on caregiver's assessment.

Activities	Present	Past	Percent decrease	Number	*P* value
Basic ADL					
Dressing	3.05 ± 0.83	4.00	23.7	39	*P* < 0.01
Bathing	3.28 ± 1.00	4.00	17.9	39	*P* < 0.01
Grooming	3.51 ± 0.68	4.00	12.2	39	*P* < 0.01
Toilet	3.59 ± 0.82	4.00	10.3	39	*P* < 0.01
Feeding	3.92 ± 0.27	4.00	1.9	39	ns
Physical ambulation	3.92 ± 0.27	4.00	1.9	39	ns
Instrumental ADL					
Responsibility for own medications	0.58 ± 0.76	3.00	80.7	38	*P* < 0.01
Ability to handle finances	0.59 ± 0.93	2.92 ± 0.36	79.6	37	*P* < 0.01
Mode of transportation	0.92 ± 1.14	2.97 ± 0.16	69.1	37	*P* < 0.01
Food preparation	0.86 ± 0.85	2.39 ± 0.96	64.2	28	*P* < 0.01
Shopping	1.09 ± 0.71	2.62 ± 0.78	58.4	34	*P* < 0.01
Ability to use telephone	1.85 ± 1.04	2.97 ± 0.16	37.9	39	*P* < 0.01
Housekeeping	1.35 ± 0.85	2.15 ± 1.13	37.0	34	*P* < 0.01
Laundry	1.74 ± 1.02	2.56 ± 0.89	31.9	27	*P* < 0.01
Advanced ADL (common AADL)					
Participation in a meeting	0.84 ± 0.99	3.00	71.9	32	*P* < 0.01
Giving advice to family	0.92 ± 0.95	2.89 ± 0.46	68.2	37	*P* < 0.01
Reading a newspaper	1.32 ± 1.14	3.00	56.1	38	*P* < 0.01
Shopping on special occasions	1.38 ± 0.86	2.78 ± 0.63	50.5	37	*P* < 0.01
Socializing with others	l.54 ± 0.98	3.00	48.6	35	*P* < 0.01
Watching TV	1.82 ± 1.05	3.00	39.3	39	*P* < 0.01
Taking a walk	2.26 ± 0.92	3.00	24.6	23	*P* < 0.01
Care of a grandchild	2.64 ± 0.50	3.00	12.1	11	*P* < 0.05

**Table 3:** Difference of present activities of daily living (ADL) evaluated by patients and caregivers.

Activity	Caregiver evaluation	Self-evaluation	Level of inconsistency^∗^	Number	*P* value
Instrumental ADI					
Responsibility for own medication	0.58 ± 0.76	1.75 ± 1.17	3.02	31	*P* < 0.01
Ability to handle finances	0.59 ± 0.93	1.73 ± 1.07	2.93	31	*P* < 0.01
Mode of transportation	0.92 ± 1.14	1.72 ± 1.23	1.87	35	*P* < 0.01
Food preparation	0.86 ± 0.85	1.65 ± 1.13	1.92	24	*P* < 0.01
Shopping	1.09 ± 0.71	1.81 ± 1.11	1.66	26	*P* < 0.01
Ability to use telephone	1.85 ± 1.04	2.59 ± 0.55	1.40	37	*P* < 0.01
Housekeeping	1.35 ± 0.85	2.19 ± 1.11	1.62	26	*P* < 0.01
Laundry	1.74 ± 1.02	2.42 ± 0.93	1.39	23	*P* < 0.01
Advanced ADL (common AADL)					
Participation in a meeting	0.84 ± 0.99	1.52 ± 1.33	1.81	25	*P* < 0.01
Giving advice to family	0.92 ± 0.95	1.38 ± 1.06	1.50	24	*P* < 0.05
Reading a newspaper	1.32 ± 1.14	2.37 ± 1.02	1.80	37	*P* < 0.05
Shopping on special occasions	1.38 ± 0.86	1.26 ± 1.03	0.91	29	ns
Socializing with others	1.54 ± 0.98	2.06 ±1.24	1.34	29	*P* < 0.05
Watching TV	1.82 ± 1.05	2.26 ± 0.92	1.24	38	ns
Taking a walk	2.26 ± 0.92	2.42 ± 0.90	1.07	19	ns
Care of a grandchild	2.64 ± 0.50	2.75 ± 0.50	1.04	4	ns

^∗^Level of inconsistency was calculated by dividing the patient's self-evaluation score by caregivers evaluation score.

**Table 4:** Support provided by family caregivers to maintain advanced activities of daily living (AADL) of patients.

Activities	Number of activities	Number of support	Rate of support	Classification of support (number for each category)				
				Instrumental	Informational	Appraisal	Emotional	Reminding
Common AADL								
Shopping in special occasions	33	68	2.03 ± 0.92	28	11	1	5	23
Participation in a meeting	16	31	1.94 ± 1.00	12	3	0	4	12
Socializing with others	29	31	1.07 ± 0.88	8	1	0	4	18
Taking a walk	21	20	0.95 ± 0.86	7	1	0	5	7
Reading a newspaper	27	14	0.52 ± 0.80	2	2	0	6	4
Watching TV	35	17	0.49 ± 0.85	2	3	0	7	5
Care of a grandchild	11	2	0.18 ± 0.60	1	0	0	0	1
Giving advice to family	21	0	0.0	0	0	0	0	0
Subtotal common ADLs	193	183	0.90 ± 0.74	60	21	1	31	70

Leisure activities								
Going to watch a movie and/or a concert	13	32	2.46 ± 0.52	13	1	0	7	11
Making a trip	15	35	2.33 ± 0.72	14	1	1	6	13
Sports	4	9	2.25 ± 1.50	3	1	0	2	3
Going for a calligraphy lesson, and so forth	13	26	2.00 ± 0.71	10	3	0	1	12
Continuation of work	4	7	1.75 ± 0.96	2	3	0	0	2
Playing games	8	9	1.13 ± 0.99	3	0	0	2	4
Playing a musical instrument, singing	4	4	1.00 ± 0.82	1	0	0	1	2
Care of a pet	3	2	0.67 ± 1.15	1	0	0	0	1
Gardening	11	4	0.36 ± 0.92	1	1	0	1	1
Other	5	13	2.60 ± 0.89	4	2	0	3	4
Subtotal of leisure activities	80	141	1.66 ± 0.92	52	12	1	23	53

Total	273	324	1.31 ± 0.84	112	33	2	54	123
